# Feasibility of Group Telerehabilitation for Older Adults: A Quality Improvement Project

**DOI:** 10.5195/ijt.2024.6651

**Published:** 2025-01-15

**Authors:** Emily M. Hudson, Stephanie P. Bazal, Michelle R. Rauzi, Hillari S. N. Olson, Melissa J. Ludescher, Christine Interrante, Molly Lahn, Estee Berg, Howard A. Fink, Jennifer P. Wisdom, Allison M. Gustavson

**Affiliations:** 1 Veterans Affairs Health Services Research and Development Center for Care Delivery and Outcomes Research, Minneapolis Veterans Affairs Health Care System, Minneapolis, MN USA; 2 Physical Medicine and Rehabilitation, Minneapolis Veterans Affairs Health Care System, Minneapolis, MN, USA; 3 Denver/Seattle Center of Innovation for Veteran-Centered and Value Driven Care, Rocky Mountain Regional Veterans Affairs Medical Center, Aurora, CO, USA; 4 Physical Therapy Program, Department of Physical Medicine and Rehabilitation, University of Colorado Anschutz Medical Campus, Aurora, CO, USA; 5 Geriatric Research Education and Clinical Center, Veterans Affairs Health Care System, Minneapolis, MN, USA; 6 Department of Medicine, University of Minnesota, Minneapolis, MN, USA; 7 Wisdom Consulting, New York, New York, NY, USA; 8 Rehabilitation & Engineering Center for Optimizing Veteran Engagement & Reintegration, Minneapolis Veterans Affairs Health Care System, Minneapolis, MN, USA; 9 Department of Medicine, University of Minnesota, Minneapolis, MN, USA

**Keywords:** Fit for Life, Group rehabilitation, Physical therapy, Telerehabilitation, Veterans

## Abstract

A growing body of evidence suggests group rehabilitation may empower patients to achieve functional goals by leveraging social connectivity. From previous work, we adapted an in-person group for older Veterans to a telerehabilitation group called Fit for Life. The current quality improvement project aimed to evaluate the feasibility of implementing Fit for Life. Eligible Veterans lived in the community and were at risk for falls or hospitalization per functional performance measures. We used convergent parallel mixed methods approach in the evaluation. Eighteen Veterans ≥55 years old (all male, mean 77 years, 39% rural) received a referral to Fit for Life, two did not participate. We analyzed and integrated adaptations posed by clinicians and Veterans in real-time to enhance access to and participation in Fit for Life. Future work will explore clinical effectiveness, tools to identify patients most likely to benefit, and care delivery structures that integrate telerehabilitation groups for older Veterans.

The Veterans Health Administration (VHA) has a long history of physical therapy utilization through telehealth to enhance continuity of care, augment timely initiation of services, and reach Veterans living in rural areas (Cowper-Ripley et al., 2019; Darkins et al., 2008; Hale-Gallardo et al., 2020; Hatzakis et al., 2003; Kreider et al., 2022; Scholten et al., 2019). VHA's support of physical therapy telerehabilitation was ahead of other healthcare systems at the onset of the COVID-19 pandemic because the technology infrastructure, processes, and supports were already in place to shift individual sessions from in-person to telerehabilitation. However, most VHA physical therapy group programs immediately shut down due to limited guidance on how to adapt such group programs to the virtual space.

Compared with individual therapy, a growing body of evidence suggests in-person group rehabilitation is promising in empowering patients to achieve functional goals by leveraging social connectivity (Gustavson et al., 2021; Rauzi, Abbate, et al., 2023; Rauzi, Mealer, et al., 2023; Yalom & Leszcz, 2020). As such, it is important to understand how to best adapt and evaluate in-person rehabilitation groups to telerehabilitation groups. As an initial step, we convened a team to develop guidance for practice considerations for telerehabilitation groups (Gustavson et al., 2021). From this work, we adapted and modified an in-person group for older Veterans to the “Fit for Life” telerehabilitation group, which is an 8-session program focused on functional strength, balance, and goal setting.

The purpose of this quality improvement project was to evaluate the feasibility (Proctor et al., 2011) of implementing the Fit for Life program as an outpatient physical therapy service at the Minneapolis Veterans Affairs Health Care System (MVAHCS), an entity of VHA. We used an implementation framework to assess reach, effectiveness, and implementation (Glasgow et al., 1999, 2019; Kwan et al., 2019). This information is necessary to inform VHA operational partners of Veterans’ experiences and the resources needed to sustain and deliver such care.

## Methods

We conducted a quality improvement project to evaluate the feasibility of Fit for Life in prospectively enrolled Veterans from March 2022 to September 2023. We used a convergent parallel mixed-methods design (Schoonenboom & Johnson, 2017) to collect quantitative and qualitative data guided by the Reach, Effectiveness, Adoption, Implementation, and Maintenance framework (RE-AIM; Glasgow et al., 2019). In this quality improvement evaluation, we focused on *reach, effectiveness,* and *implementation* with operationalized definitions as follows. *Reach* refers to the proportion of the target population referred (Glasgow et al., 2019). *Effectiveness* refers to how an intervention impacts an individual's health outcomes. *Implementation* refers to the adaptations to the intervention (i.e., Fit for Life) to increase Veteran participation and strategies to help physical therapists deliver the intervention optimized for Veteran participation. A co-design approach was implemented to inform real-time adaptions to the Fit for Life program (Woodward et al., 2023). Co-design projects involve collaboration between project staff, service users (Veterans), and key informants (physical therapists) to identify problems and develop solutions (Singh et al., 2023). The Minneapolis VA Institutional Review Board designated this project as a quality improvement project. We followed the Standards for Quality Improvement Reporting Excellence 2.0 (SQUIRE) for this publication (Ogrinc et al., 2016).

### Setting & Sample

Fit for Life was provided through the MVAHCS, which serves Veterans in Minnesota and Western Wisconsin through the main campus in Minneapolis, as well as nine Community Based Outpatient clinics (CBOCs). Veterans were eligible for Fit for Life if they were ≥55 years old, living in the community, and at risk for falls or hospitalization per routine clinical functional performance measures (e.g., 5 times sit-to-stand, 4-stage balance test, Patient Specific Functional Scale; Bohannon, 2006; Bohannon et al., 2007; Jones et al., 1999; Rossiter-Fornoff et al., 1995; Stratford et al., 1995). The Veterans were referred from either inpatient or outpatient settings for an initial evaluation with an outpatient physical therapist. During the evaluation, the physical therapist provided education on the Fit for Life program as an option for care and, if the Veteran was agreeable, referred them to self-directed scheduling to complete scheduling for group telerehabilitation. Following completion of the group, Veterans were scheduled to return to their referring therapist for an individual session to repeat the functional performance measures. To gather clinician perspectives, outpatient and inpatient physical therapists at the MVAHCS participated in a semi-structured focus group that was held during a regularly scheduled meeting. All physical therapists could refer to Fit for Life, regardless of setting.

### Fit for Life Intervention

One physical therapist (author, SPB) delivered the Fit for Life group telerehabilitation program. At the first group visit, the Fit for Life physical therapist reviewed the telehealth group agreement with participants, which outlined expectations for group participation, such as privacy and respect for persons in the group. Sessions followed a protocol (Appendix A) that progressed Veterans through a series of exercises focused on improving function and activities of daily living. Exercises targeted different muscle groups, such as lower extremity (quadriceps and gluteals) and upper extremity muscles (biceps and deltoids), to enhance independence with activities such as getting out of a chair and carrying laundry or groceries.

To individualize the experience during group sessions, the Fit for Life physical therapist monitored Veterans and demonstrated modifications for exercises as needed, such as how to perform while sitting or standing and encouraging those with poor balance to hold onto the back of a chair or counter. Veterans were monitored in-session to ensure they were at an optimal level of exertion. To determine exertion level, Veterans were asked to subjectively rate their self-perceived exertion level based on a standardized scale (Borg, 1998) throughout the Fit for Life sessions. The physical therapist would then adjust and tailor the exercises to ensure Veterans were at a moderate level of exertion. Upon completion of the Fit for Life program, participants were scheduled to attend a virtual or in-person discharge appointment with the physical therapist who referred them to Fit for Life to determine on-going rehabilitation needs. A shared decision-making model (Elwyn et al., 2012) was used to determine if the Veteran was appropriate for discharge to a home exercise program or if they needed additional, individual visits to achieve remaining or new functional goals.

### Quantitative Data Collection

We collected quantitative data to evaluate *reach*, *effectiveness*, and *implementation*. *Reach* was measured by the number of Veterans referred and completed at least one session. In addition, we collected Veteran characteristics, reasons for refusal, participation rates (e.g., attendance, percentage of completed sessions, no-shows), and time from referral to the first appointment. Veteran characteristics were collected from the electronic health record (EHR) and included age, sex, race, ethnicity, rurality, body mass index, and living environment. *Effectiveness* was assessed using patient-level outcome measures collected during routine care as part of the initial evaluation and discharge completed by referring physical therapists; the type of measures used varied by clinician based on their preferences and clinical decision-making style. Outcome measures were extracted from the EHR. *Implementation* was measured by physical therapist reported time spent on technology issues during the session and documentation of the group session.

### Qualitative Data Collection

We collected qualitative data for *reach*, *effectiveness,* and *implementation* through interviews (Veterans) and focus groups (MVAHCS physical therapists). With Veterans, we completed phone-based, individual, semi-structured interviews following completion of the Fit for Life group telerehabilitation program. Qualitative open-ended interviews focused on Veteran's perceptions of the Fit for Life program (See Appendix B for interview guides). We also included questions to prompt feedback about Fit for Life processes and elicited ideas for adaptation and improvement. Additionally, qualitative interviews were completed with Veterans who were referred to Fit for Life but declined to participate to understand potential barriers and suggested adaptations to overcome challenges to participation (See Appendix C for interview guides).

All Veteran interviews were conducted by one member of the research team (EMH). To ensure trustworthiness of qualitative interview data, EMH was trained by author JPW in conducting qualitative interviews. JPW is a qualitative methodologist consultant, who provided training on the qualitative interview process and facilitated practice interviews to provide targeted feedback on areas for improvement. Veteran interviews were not recorded or transcribed because it is not considered standard practice to record conversations with Veterans about program evaluation and improvement. Therefore, instead of completing verbatim transcripts, a summative approach was used (Halcomb & Davidson, 2006). First, the interviewer (EMH) took detailed field notes during the interviews. To ensure trustworthiness, EMH would briefly summarize the notes to participants during the phone call to verify accuracy of notes. Then, immediately after the interview, the field notes were compiled into a detailed summary.

With MVAHCS physical therapists, we conducted two waves of semi-structured focus groups. Each focus group occurred during virtual, one-hour team meetings, facilitated by co-author SPB with field notes taken by co-author AMG. Each wave of focus groups included one session with inpatient physical therapists and one session with outpatient physical therapists. The first wave of focus groups were conducted in December 2022 during the early phases of Fit for Life implementation. The aims of the first wave were to identify physical therapists’ perspectives of the program's effectiveness, understand gaps in the referral processes, and co-create adaptations to increase the reach and implementation of the program. We conducted the second wave of focus groups in August 2023 to better understand low referral rates. The second wave of focus groups questions elicited physical therapists’ perceptions of access, barriers to both the referral process and Veteran participation, and adaptations for further improvement in reach and implementation.

**Figure 1:**
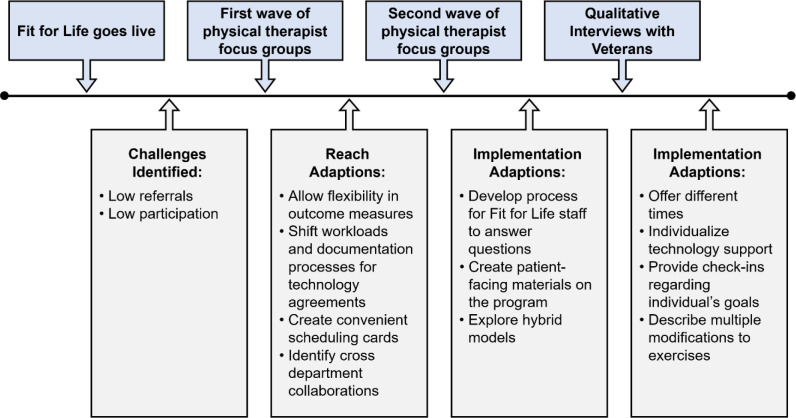
Fit for Life Program Evaluation Data Collection Timeline and Adaptations

Focus groups were completed during a standardized team meeting, which were not allowed to be recorded due to Union Stipulations. Therefore, instead of completing verbatim transcripts, a summative approach was used (Halcomb & Davidson, 2006). First, a member of the research team (EMH, AMG) took detailed field notes during the focus groups. Then, immediately after the focus groups, a detailed summary was completed. To ensure trustworthiness of the data collected, the detailed summaries were reviewed for accuracy by the facilitator of the focus groups (SPB). A timeline of data collection is presented in Figure 1.

### Quantitative Data Analysis

We calculated descriptive statistics for all Veteran characteristics and reported using counts (%) and mean (standard deviation [SD]). For analysis of functional performance measures pre- and post- intervention, we calculated the proportion of participants with clinically meaningful improvements in each respective measure. Clinically meaningful improvement was expressed as a change in performance that exceeded the minimally important difference established for a measure (Guralnik et al., 2020, p. 2014). All analyses were performed using Microsoft Excel (Version 2021).

### Qualitative Data Analysis

We completed three qualitative analyses using a rapid, directed content approach (Beebe, 2014; Hsieh & Shannon, 2005). Summative data from the first and second waves of focus groups were analyzed separately to inform adaptations or modifications to the program or processes in real-time for maximal impact on clinician and Veteran outcomes. The third analysis included summative data obtained from Veteran interviews. All data were analyzed by three members of the research team (AMG, EMH, SPB), which involved compiling data into separate matrices for each wave of data analysis that were organized with the participants in rows and questions in columns (Meyer & Avery, 2009). Then deductive analysis guided by the RE-AIM framework was completed and evaluated for themes within each construct of the RE-AIM framework. To ensure trustworthiness, data analysis was completed independently by two members of the data analysis team and reviewed for consistency. The team met immediately following interviews and focus groups to summarize the field notes and discuss the implications of suggested adaptations on real-time modifications to the program or processes. Additionally, a monthly team meeting occurred to discuss emerging themes and inform potential adaptations. As part of the co-design process, summaries of the analyses were generated monthly to share with clinical staff and operational leadership to inform adaptations and impact in real-time.

## Results

Eighteen Veterans were referred to Fit for Life. All 18 Veterans met eligibility criteria. Referred Veterans averaged 76.9 years of age, all were white, male, and most lived at home with a friend or family member (87%). See Table 1 for complete demographic information. Thirty outpatient and 15 inpatient physical therapists participated in Wave 1 and Wave 2 focus groups, respectively. Physical therapists worked in a variety of settings including inpatient rehabilitation, acute medicine, cardiopulmonary rehabilitation, outpatient orthopedics and neurorehabilitation.

**Table 1:** Veteran Demographics and Clinical Characteristics of Those Referred to Fit for Life

Variable	Mean ± SD or Frequency (%)		
	Total (n=18)	Participating (n=16)	Declining (n=2)
Age, mean ± SD	76.9 ± 5.2	76.8 ± 5.5	77.5 ± 0.5
Sex, No. (%)			
Male	18 (100)	16 (89)	2 (11)
Race, No. (%)			
White	17 (94)	15 (83)	2 (11)
Black or African American	1 (6)	1 (6)	0 (0)
Ethnicity, No. (%)			
Not Hispanic or Latino	18 (100)	16 (89)	2 (11)
Rurality, No. (%)			
Urban	11 (61)	10 (56)	1 (6)
Rural	7 (39)	6 (33)	1 (6)
BMI, Mean ± SD	30.8 ± 4.3	30.8 ± 4.2	30.5 ± 5.4
Living environment, No. (%)			
House	12 (67)	10 (56)	2 (11)
Apartment	3 (17)	3 (17)	0 (0)
Assisted living or senior living	2 (11)	2 (11)	0 (0)
Social support, No. (%)			
Lives alone	1 (6)	1 (6)	0 (0)
Lives with family or friends	15 (83)	13 (72)	2 (11)
Receives social services	2 (11)	2 (11)	0 ()
Recent hospitalization, No. (%)	3 (17)	3 (17)	1 (6)
Post-acute care utilization (short-term rehabilitation	2 (11)	2 (11)	0 (0)

*Note.* SD=standard deviation; BMI=body mass index

### Reach

Eighteen Veterans were referred to Fit for Life and, of those, two declined participation due to other medical needs and a preference for in-person services. Sixteen Veterans attended one session, completing a mean of six out of eight planned sessions. Five Veterans had one no-show and one Veteran had two no-shows. The average duration from referral to first appointment was 18 ± 13 days.

Despite all referred Veterans being eligible and most Veterans expressing interest in participating, only 14 Veterans had been referred within the first 16 months. Which was determined by the Fit for Life team as having low referral rates. To better understand the low referral rates, a second focus group was conducted. Results of the second focus group confirmed that physical therapists identified low referral rates as challenges to Veteran's accessing and potentially benefiting from the program. Physical therapists spoke about their current referral decision making process as identifying Veterans as appropriate for the program if they were sedentary but internally motivated. Physical therapists described their existing referral process which included an initial one-on-one evaluation with assessment of functional performance outcomes, completion of group telehealth agreement, and provision of patient scheduling details for the group Fit for Life program. Physical therapists reported that the Fit for Life referral process did increase their workload.

To increase referrals and reduce workload burden, physical therapists suggested ways to enhance access to the program through different strategies. We implemented four adaptations following the first wave of focus groups. First, to reduce workload during referral process, we allowed physical therapists to assess and evaluate the Veteran's initial eligibility for Fit for Life based on functional performance measures used within their individualized practice (i.e., not a standardized measure). Second, we moved additional screening for technology needs and safety with virtual care delivery (i.e., balance issues during exercises appropriateness) to the Fit for Life physical therapist. As part of this, we created a documentation process, using a guided template, to allow the conduct of the telehealth agreement to transition from the referring physical therapists to the Fit for Life physical therapist.

Third, we created a convenient scheduling card containing program and referral information. Scheduling cards were placed in accessible locations—identified by the physical therapists—within the outpatient and inpatient physical therapy workstations for physical therapists to hand-out to referred Veterans. Finally, physical therapists suggested it would be beneficial for Fit for Life staff to develop partnerships with existing interdisciplinary programs outside the general inpatient or outpatient services that provide some rehabilitation services but may benefit from additional participation options. We are in the process of exploring this option with our inpatient rehabilitation units and cancer rehabilitation programs.

### Effectiveness

Physical therapists used a variety of functional performance outcome measures to evaluate Veteran response to the Fit for Life program. Twenty different functional performance measures were collected to assess constructs of functional mobility, activities of daily living (ADLs), gait, balance, and fall risk (Table 2). Overall, all participants were tested with at least one outcome measure, and 12 out of 16 (75%) had both pre- and post- data for at least one outcome measure. Fifteen out of 16 (93%) participants were tested for at least one functional mobility outcome, most commonly the 30-second sit-to-stand (50%). The next most frequently assessed construct was balance (13/16, 81%), with the most common balance test being the 4-stage balance test (9/16, 56%). Out of the 12 participants with pre- and post-data on at least one outcome measure, 8 (75%) showed clinically meaningful change specific to the outcome measure used. For example, if a Veteran had both pre- and post- 30 second sit to stand data, the clinically meaningful change for that test is an increase of two repetitions (Gill et al., 2022; Zanini et al., 2019). The most common area of improvement was seen on the 5-time sit-to-stand (n=3) and the 4-stage balance test (n=3), followed by the Patient Specific Functional Scale (n=2).

**Table 2:** Effectiveness Data Regarding the Frequency of Use, Complete Data, and Clinically Meaningful Improvement on Functional Outcome Measures

Cluster Type and Outcome Measures	Frequency of Use N (%)	Frequency of Complete Data N (%)	Patients with Clinically Meaningful Improvement N (%)
**Functional Mobility**	16 (100)	12 (75)	2 (12)
Patient-Reported Outcomes Measurement Information System: Mobility Tool (PROMIS Mobility) 30 sec sit to stand Floor Transfer Short Physical Performance Battery (SPPB) Timed Up and Go (TUG) 5x sit to stand			
**Activities of Daily Living**	7 (44)	2 (12)	2 (12)
Patient Specific Functional Scale (PSFS) Parkinson's Disease Questionnaire (PDQ-39)			
**Gait**	7 (44)	5 (31)	3 (19)
Gait Speed Functional Gait Assessment (FGA) 6-minute walk test (6MWT) 2-minute step test (MST)			
**Balance**	13 (81)	6 (37)	3 (19)
Mini Balance Evaluation Systems Tests (mini-BESTest) Berg Balance Scale (BBS) Single Leg Stance Test (SLS) 4-Stage Balance Test (SBT) Turning 360 degrees			
**Fall Risk**	1 (6)	0 (0)	N/A
Missouri Alliance for Home Care's (MAHC) fall risk assessment			

*Note.* Patients may have been assessed on or shown clinically meaningful improvement on more than one outcome measure across cluster types.

Interviews with 12 Veterans who participated in Fit for Life indicated positive experiences centered around improving or maintaining their physical health. Several Veterans expressed the importance of maintaining an exercise routine to prevent further deterioration of mobility due to health conditions. Fit for Life was perceived to be beneficial in helping develop an exercise routine in their life. For example, one Veteran explained that while there is no cure for Parkinson's Disease, the exercises completed during Fit for Life helped reduce their symptoms and encouraged them to move in their daily life. Another Veteran expressed that, despite being in a group setting, the exercises were tailored to their needs and helped them improve their mobility. Additionally, one aim of the first wave of focus groups was to inquire about physical therapist's perceptions of the program. Overall, physical therapists perceived the program as valuable for maintaining or improving Veteran's mobility.

### Implementation

In terms of process indicators for Fit for Life, clinicians spent less than five minutes per group telerehabilitation session on resolving technology issues and less than ten minutes documenting on all participants following the session. Implementation was further evaluated based on clinical data and participants' perceptions of adaptions that could improve Fit for Life. Veterans suggested adaptations centered around flexibility in care delivery, technology needs, and individualized programming in the group context. Veterans expressed a need for rehabilitation programs to offer flexible scheduling and offer a variety of modalities (i.e., in-person, group telerehabilitation, individualized telerehabilitation). In addition, Veterans explained that the group sessions were more difficult to attend because there was less flexibility with the days and times offered. While most Veterans described the convenience of participating in the program via video, some Veterans experienced difficulties such as inadequate screen sizes on their phone or tablet. Some Veterans did not own a device with video capabilities. Finally, Veterans expressed a need for more individualization in the group context. Suggestions for more personalization approaches included checking-in with the Veteran's individual goals, providing modifications based on their functioning levels, and providing reminders not to overexert themselves during exercises. Based on Veteran feedback, the following adaptions were implemented. First, individualized support was provided during the initial technology assessment to adjust video settings and optimize participation. Second, the Fit for Life physical therapist provided additional verbal prompts to explain exercises, tailored the exercises to each participant's goals, and demonstrated multiple modifications for each exercise aligned with the physical abilities of each participant. Third, morning and afternoon options were initially made available to increase participation.

Physical therapists identified low Veteran participation as a challenge to implementation of Fit for Life, despite adaptations to the referral process. During the second wave of focus groups, physical therapists expressed that the scheduling card and updated documentation processes implemented following the first wave of focus groups improved ease of referrals. However, physical therapists explained that Veterans were unaware of the program, and thus, Veterans had insufficient information to evaluate if the program was appropriate for their needs and warranted participation. Physical therapists recommended improving accessibility of public-facing information for Veterans to learn about the Fit for Life program and including statistics on the success rates. Referring physical therapists were also concerned that solely a virtual approach may not be appropriate for all Veterans because of device availability and technological proficiency, and the potential need for occasional hands-on assessments and treatments to ensure safety. As a result, physical therapists suggested Fit for Life offer a combination of in-person and virtual options. To address this, we created a process for communicating with Fit for Life staff regarding Veteran and clinician questions or concerns. We are in the process of creating an infographic for Veterans and are in discussion with clinical leadership on how a hybrid model may work.

## Discussion

The Fit for Life group telerehabilitation program feasibly recruited and retained older Veterans with most completing >75% of the 8-week program. Veterans and physical therapists indicated positive experiences and perceived improvements in health outcomes. Suggested adaptations were integrated into ongoing program development to enhance reach and implementation.

There is growing evidence to support the feasibility and acceptability of group telerehabilitation in older adults with traumatic brain injury, spinal cord injury and other medically complex conditions, despite challenges related to flexibility with synchronous attendance (Baehr et al., 2023; Boulos et al., 2024; Rauzi, Abbate, et al., 2023; Rauzi, Mealer, et al., 2023). We found similar results in our qualitative evaluation as participants explained that group sessions were more difficult to attend because there was less flexibility with the days and times that they were offered. The physical therapists identified challenges and opportunities based on referral pathways as upstream contributors to accessing group telerehabilitation. Future research or quality improvement projects are needed to create tools to screen patients for different rehabilitation delivery modalities, as well as to develop shared decision aids to identify the right modality of rehabilitation to meet their preferences and needs.

To optimize the reach and implementation of Fit for Life, we integrated input from physical therapists and eligible Veterans in a form of co-design that informed our quality improvement activities. The lack of co-design approaches to inform strategies and program modifications in real time remains a gap in the rehabilitation literature (Jesus et al., 2023). Consequently, there is limited guidance for new programs on how to capture and act on the experiences shared by individuals delivering or receiving health care services. Thus, we sought to initiate action based on feedback from key informants (physical therapists, Veterans) to enhance the reach and implementation of Fit for Life. Future work will integrate more formal co-design strategies and evaluate the impact of iterative changes to identify enablers, barriers, and unintended consequences.

Findings from this project support the feasibility of group telerehabilitation as an option for care delivery. Such an approach is clinically relevant, especially for patients who are immunocompromised, have limited mobility, or live in rural areas because telerehabilitation can help maintain health and safety and improve access to services. Participants had positive experiences and demonstrated clinically meaningful improvements in functional ability following Fit for Life. The largest barrier to the success of the Fit for Life program was the small number of Veterans reached and who participated. This could be addressed by creating greater awareness and visibility of the program to Veterans, physical therapists, and other referring providers, and by partnering with other clinical programs (e.g., cancer rehabilitation, inpatient rehabilitation units).

## Limitations

There were some limitations to our quality improvement project. First, we were unable to objectively evaluate clinical effectiveness of the program due to the lack of a standardized clinical assessments. Future quality improvement projects or research studies should evaluate program effectiveness by standardizing outcome measures across inpatient and outpatient rehabilitation care. Despite limitations in fully evaluating effectiveness, our large health care system is invested in evaluating this program further. A second limitation is the constrained evaluation on how adaptations to the program and referral processes influenced implementation outcomes. Finally, because all study participants were male, the results of this project may not be generalizable to other populations. As a quality improvement project, the results are not generalizable to other healthcare systems. However, this project serves as an exemplar for how to develop, implement, evaluate, and continually adapt a group telerehabilitation program.

## Conclusions

This quality improvement project evaluated the reach, effectiveness, and implementation of the Fit for Life group telerehabilitation program. Findings included salient barriers to participation and the referral process. Our co-design approach facilitated shareholder-identified solutions and practical adaptions to improve access to the Fit for Life program. Fit for Life can address gaps in rehabilitation for vulnerable populations (e.g., rural patients) and, thereby, contribute to post-pandemic efforts to recreate new, more flexible, patient-centered healthcare systems.

## Appendix A Fit for Life Session Overview

Fit for Life Overview **Eligibility Criteria:** Must be able to connect to VA video connect test screen, stand feet together without assistance for 10 seconds per 4 Stage Balance Test, walk 25’ with or without support per PROMIS Physical Function with Mobility Aid, and be in Preparation stage of the Stages of Change Algorithm. **Technology Required:** Patient must have access to a video device with reliable internet access

Session 1 **Focus:** Floor transfers (core, strength) **Warm-up:** High knees marching [1–2 min], sit to stands [1–2 min], [2–3/10 Rate of Perceived Exertion] **Exercises:** 3–5 rounds: standing to ½ kneeling [1 min], ½ kneeling to quadruped [1 min], bird dog [1 min], and dead bugs [1 min] **Post-session Goals:** Tailor post-session exercises for each patient's needs, direct patients to online resources

Session 2 **Focus:** Laundry/Groceries (upper body strength) **Warm-up:** High knees marching [1–2 min], side stepping [1–2 min], side-to-side reaches + mini squat vs lateral weight shifts, [2-3/10 Rate of Perceived Exertion] **Exercises:** 3–4 rounds: squat down/reach up [2 min], chair/countertop plank [2 min with breaks], active upper extremity strength/shoulder row + overhead reach [2 min], 4) squat/reach down to left turn to reach up to right [2 min] **Post-session Goals:** Tailor post-session exercises for each patient's needs, direct patients to online resources

Session 3 **Focus:** Walking (aerobic activity) **Warm-up:** Seated hamstrings stretch on each leg twice [30 sec], knee flexion [2 mins], heel-toe raises [2 mins] **Exercises:** 3–5 rounds: hip abduction [30 sec each side], controlled arm swings [30 sec each side], lunges [1 min], marching in place [3 mins] **Post-session Goals:** Tailor post-session exercises for each patient's needs, direct patients to online resources

Session 4 **Focus:** Getting out of a chair (core, strength) **Warm-up:** High knees marching [2 mins], side stepping [2 mins], modified jacks [2 mins] **Exercises:** 2–3 rounds: Chair stands [1 min], chair dips [1 min], wide legged squats [1 min], modified lunges [1 min] **Post-session Goals:** Tailor post-session exercises for each patient's needs, direct patients to online resources

Session 5 **Focus:** Cleaning/dishes (picking things up off the ground) **Warm-up:** marching in place [1 min], standing trunk rotation [1 min], arm circles [1 min], seated forward flexion [1 min], seated trunk rotation [1 min], cross body arm stretch [1 min] **Exercises:** Forward/backward steps [1 min], toe raises [1 min], overhead UE flexion [1 min], hip hinge/modified deadlift [1 min] cross body reach with alternating step [1 min], lateral stepping [1 min] forward alternating lunge [1 min], bend over row [1 min] **Post-session Goals:** Tailor post-session exercises for each patient's needs, direct patients to online resources

Session 6 **Focus:** Keeping steady while moving (balance) **Warm-up:** trunk movements [5 min], high knees marching [5 min] **Exercises:** 4 rounds: squats [1 min], sideways walking [1 min], heel/toe walking [1 min], sit to stands [1 min] **Post-session Goals:** Tailor post-session exercises for each patient's needs, direct patients to online resources

Session 7 **Focus:** Outdoor activities (gardening, mowing, shoveling) **Warm-up:** high knees marching [5 mins], multidirectional arm circles [5 mins] **Exercises:** Squats [1 min], lunges [1 min], wall push-ups [1 min], half-kneeling with rotation [1 min], marching [2–3 mins], seated stretching rotation [1–2 mins], seated stretching upright cat/cow [1–2 mins] **Post-session Goals:** Tailor post-session exercises for each patient's needs, direct patients to online resources

Session 8 **Focus:** Bringing it all together **Warm-up:** Marching in place [1 min], alternating lateral sidestep [1 min] **Exercises:** reaching above head side stretch [30 sec/side], chair dip [1 min], single leg stance [1 min], repeated sit to stand [15 reps] **Post-session Goals:** Tailor post-session exercises for each patient's needs, direct patients to online resources

Discharge Plans **Individual evaluation:** evaluation of functional performance outcomes and tailor discharge recommendations to patient's individual needs **Referrals:** Patient is referred to community based and/or technology-based exercise programs

## Appendix B Veteran Interview Guide for Those Who Participated in Fit for Life

**Table:**

		Question
**0.**	**Grand Tour**	Can you tell me a little bit about your experience receiving physical therapy over the video?
**1.**	**Veteran Satisfaction with the Fit for Life Program**	What was your overall impression of the program? What did you like? What did you not like? Do you have any suggestions to make this program better?
**2.**	**Veteran Satisfaction with Referral Process**	What was your experience scheduling the video therapy sessions? What parts went well? What parts were challenging? What questions did you have when you first heard about the program?
**3.**	**Perceptions of Access**	What do you think would prevent another veteran from participating in this program? What suggestions do you have to encourage more Veterans to participate in this program?
**4.**	**Wrap-up**	*Optional: If you could say one thing about the program, what would you want other Veterans to know?*

## Appendix C Veteran Interview Guide for Those Who Were Referred to Fit for Life but Declined Participation

**Table:**

	**Question**
**1. Overview**	In the past, a provider talked to you about a “Fit for Life” program where you could receive physical therapy in a group over video in your own home. Would you like an explanation about this program?
**2. Perception of access**	What were your impressions about the program? What questions did you have about the program?
**3. Barriers to Participation**	Can you share a little more about why you chose not to participate? What concerns did you have? What would you have needed to participate?
**4. Suggestions**	What suggestions do you have to improve how this is offered to veterans? What resources would have been helpful to you in making your decision? Is there anything that you wish would have been explained to you?
**5. Wrap-up**	Thank you for your time and insight.
